# Vascular Endothelium as a Target of Immune Response in Renal Transplant Rejection

**DOI:** 10.3389/fimmu.2014.00505

**Published:** 2014-10-21

**Authors:** Giovanni Piotti, Alessandra Palmisano, Umberto Maggiore, Carlo Buzio

**Affiliations:** ^1^Kidney and Pancreas Transplantation Unit, Department of Clinical Medicine, Nephrology and Health Sciences, University Hospital of Parma, Parma, Italy

**Keywords:** endothelial cell antigens, angiotensin II type 1 receptor, vimentin, accommodation, regulatory T-cells, renal transplantation, antibody-mediated rejection, mTOR inhibitors

## Abstract

This review of clinical and experimental studies aims at analyzing the interplay between graft endothelium and host immune system in renal transplantation, and how it affects the survival of the graft. Graft endothelium is indeed the first barrier between self and non-self that is encountered by host lymphocytes upon reperfusion of vascularized solid transplants. Endothelial cells (EC) express all the major sets of antigens (Ag) that elicit host immune response, and therefore represent a preferential target in organ rejection. Some of the Ag expressed by EC are target of the antibody-mediated response, such as the AB0 blood group system, the human leukocyte antigens (HLA), and MHC class I related chain A antigens (MICA) systems, and the endothelial cell-restricted Ag; for each of these systems, the mechanisms of interaction and damage of both preformed and *de novo* donor-specific antibodies are reviewed along with their impact on renal graft survival. Moreover, the rejection process can force injured EC to expose cryptic self-Ag, toward which an autoimmune response mounts, overlapping to the allo-immune response in the damaging of the graft. Not only are EC a passive target of the host immune response but also an active player in lymphocyte activation; therefore, their interaction with allogenic T-cells is analyzed on the basis of experimental *in vitro* and *in vivo* studies, according to the patterns of expression of the HLA class I and II and the co-stimulatory molecules specific for cytotoxic and helper T-cells. Finally, as the response that follows transplantation has proven to be not necessarily destructive, the factors that foster graft endothelium functioning in spite of rejection, and how they could be therapeutically harnessed to promote long-term graft acceptance, are described: accommodation that is resistance of EC to donor-specific antibodies, and endothelial cell ability to induce Foxp3+ regulatory T-cells, that are crucial mediators of tolerance.

## Introduction

Over the last few decades, the practice of kidney transplantation has improved in many areas up to becoming the optimal treatment for end-stage renal disease (1). However, despite brilliantly achieving a 95% 1-year survival, long-term outcomes have not benefited from such improvements and remain unsatisfying (2). One of the major causes of late graft loss is occurrence of antibody-mediated rejection (ABMR), which current immunosuppressive regimens have mostly proven to be unable to cure (3). Another issue is the development of accelerated cardiovascular disease, which is due to the side effects inherent in the immunosuppressive drugs and, along with opportunistic infections and malignancies, represents the first cause of recipient death (4).

Allograft endothelium is the first barrier between self and non-self in vascularized solid-organ transplantation, and preservation of its integrity and functions is mandatory to ensure a prolonged survival of the graft (5). As endothelial cells (EC) express a number of antigens (Ag) that are visible by the immune system of a genetically disparate individual, donor endothelium is invariably recognized by the host immune system, and therefore, it is the first and preferential target of the allo-immune response that follows organ transplantation without an adequate immunosuppression (6).

Both naturally occurring and induced allo-antibodies directed to the Ag expressed on the membrane of EC are commonly found in renal recipients, and such antibodies, being capable of fixing the complement and damaging the tissues, are detrimental for the correct functioning of the endothelium (7). Moreover EC, besides being target of antibody-mediated response, can directly interact with allogenic T-cells by displaying not only the major histocompatibility complex (MHC) antigens but also adequate co-stimulatory molecules and adhesion proteins on their surface (8).

Nevertheless, the host immune response that follows recognition of EC allo-Ag is not necessarily destructive, in spite of graft rejection, accommodation, where not active tolerance, may operationally establish, thus fostering the endothelium to fulfill its functions (5). Endothelial regulation of blood flow and vessel permeability is paramount not only for the survival of any vascularized allograft but also for the specific depurative activities of the kidneys.

This review aims at analyzing the interplay between allograft endothelium and host immune system, and how differential unfolding of this interplay may ultimately affect the fate of the graft. A particular attention will be paid to the factors that, at the endothelial level, contribute to tipping the balance in favor of graft acceptance rather than rejection.

## Discussion

### Antibody-mediated immune response toward allograft endothelial cells

The importance of donor-specific antibodies in causing allograft rejection has progressively been uncovered, to the extent that a humoral theory of transplantation has been formulated in juxtaposition to the cellular one (9). Here, all the sets of Ag expressed on human EC that are relevant for kidney transplantation will be discussed (Table 1), along with the mechanisms of damage and accommodation.

**Table 1:** **Antibody-mediated immune response toward allograft endothelial cells**.

Type of immunity	Target Ag on EC	Preformed Ab	*De novo* Ab	C^1^ fixing Ab	Hyper- or Accel.-AR	Acute rejection	Chronic rejection	Detected by current XM	Reference
Allo-Ab	AB0	Yes	Yes	Yes	Yes	Yes	Yes	–	(10, 12, 16, 17)
	HLA	Yes	Yes	Yes	Yes	Yes	Yes	Yes	(6, 7, 18–26)
	MICA	Yes	Yes	Yes	Yes	Yes	Yes	No	(28–33)
	ECA	Likely yes	Yes	Likely yes	Likely yes	Yes	Yes	No	(35–51)
Auto-Ab	ATR1	No	Yes	No	No	Yes	Yes	No	(56, 57)
	Vimentin	No	Yes	No	No	No	Yes	No	(59, 63)

*Ag, antigens; EC, endothelial cells; Ab, antibody; C ^1^, complement; Accel., accelerated; AR, acute rejection; XM, cross-match; ECA, endothelial cell-restricted antigens; ATR1, angiotensin II type 1 receptor*.

### Endothelial Cell Antigens Target of Allo-Antibodies

#### AB0 blood group antigens

Endothelial cells highly express the AB0 blood group antigens on their surface (10). Such Ag are carbohydrates linked to glycoproteins and glycolipids and are targets of specific Ab (isoagglutinins), which occur naturally in people lacking the A and/or B antigens (11). Isoagglutinins, upon binding to A/B incompatible EC, cause hyperacute or accelerated acute graft rejection (12). AB0 compatibility has, therefore, been required for successful cadaveric transplantation; however, being the allelic variability of this system little, it does not represent a barrier for allocation of deceased donor organs. On the other hand, due to the allelic frequencies, any two individuals have roughly a 35% probability of being AB0 incompatible (AB0i), and this is an actual limitation to living renal donations (11). A first breach to the absolute requirement for AB0 compatibility emerged soon after the recognition of AB0 Ag as a barrier for solid-organ transplantation; the analysis of the outcomes showed that acceptable results were only obtained when renal grafts from A2 donors had been transplanted to non-A, i.e., 0 or B, recipients (12, 13). This donor-recipient combination proved somehow permissive because of the scarce expression of A2 Ag on EC, and the consequent low titers of anti-A2 Ab in non-A2 recipients (13). A second breach was the good results reached with AB0i heart transplants in children, who are known to express lower amount of A/B Ag and produce less Ab compared to adults (14). Finally, in 1981, the report of a successful rescue treatment for a mistakenly performed AB0i renal transplantation was published (15); the concept of removal of the isoagglutinins with plasmapheresis in order to avoid hyperacute rejection laid the basis to current practice that aims at reducing anti-A/B Ab titers before and soon after transplantation as a strategy to overcome AB0 barrier (16). Since then, kidney transplantation from AB0i living donor has become a routine practice in many transplantation centers (17).

#### Human leukocyte antigens

Major histocompatibility complex antigens, also known as human leukocyte antigens (HLA) in human beings, are highly polymorphic surface proteins whose principal function is to display Ag to T-cells for recognition and activation. Two different classes of HLA molecules exist: class I molecules are constitutively expressed by all cell types and present Ag to CD8+ cytotoxic T-cells; class II molecules, that present Ag to CD4+ helper T-cells, are commonly restricted to professional antigen-presenting cells (APC) such as dendritic cells, but, upon stimulation, can be induced onto other cell types. Human EC highly express class I and, albeit at lesser extent, also class II HLA molecules, which can be further enhanced by appropriate stimuli of inflammatory and immunologic origin (6, 7).

Apart from presenting Ag to lymphocytes, HLA molecules can themselves be recognized by an allogenic immune system, and anti-HLA Ab are produced following immunizing events like pregnancies, blood transfusions, and organ transplantation (18). Preformed anti-HLA donor-specific Ab (DSA), i.e., DSA present prior to transplantation, have long been known to be responsible for hyperacute or accelerated acute graft rejection, which is determined by mechanisms similar to those for AB0i transplantation performed without an adequate desensitization (19). Nevertheless, due to the extreme polymorphism of HLA genes, donor-recipient matching is an exceptional occurrence; therefore, unlike bone marrow transplantation, most of the kidney transplants are performed across the HLA barrier, for which a profound immunosuppression is lifelong required. In order to eliminate the risk of hyperacute rejection, laboratory cross-matching techniques have been developed, and routinely applied, to identify preformed anti-HLA Ab in the serum of recipients before transplantation; a positive donor-recipient HLA cross-match would currently halt the organ allocation in the absence of adequate desensitization (20). Moreover, anti-HLA DSA can occur *de novo* after transplantation mainly as a consequence of suboptimal immunosuppression or scarce adherence to the therapy (21). *De novo* DSA, particularly if complement-fixing Ab, is pathogenic for both acute and chronic rejection of the allograft (22); and the presence of such Ab in recipient’s serum has prospectively been linked to graft failure in several studies (23). The EC of graft peritubular capillaries (PTC) are the preferred targets of DSA so much so that microvascular inflammation is a required criterion for histopathologic diagnosis of ABMR (24), which is further underpinned by the presence of deposits of the complement fragment C4d on PTC endothelium (25, 26), and of circulating DSA (24).

#### MHC class I related chain A antigens

The description of sporadic cases of hyperacute or accelerated acute rejection of non-AB0i kidneys in recipients lacking anti-HLA DSA have urged researchers to investigate further sets of allo-antigens that might be relevant for transplantation (27). MHC class I related chain A antigens (MICA) are surface glycoproteins encoded by highly polymorphic genes located on chromosome 6 within the region of MHC genes (28). MICA, whose function is related to immune surveillance, are expressed by different types of cells including EC, but importantly neither T nor B lymphocytes; thus, current standard cross-match procedures are unable to detect anti-MICA antibodies (28). MICA have proven to be the target of complement-fixing allo-Ab that can cause hyperacute, acute, and chronic ABMR (28–32); the presence of anti-MICA DSA negatively impact short-term and long-term graft survival, albeit at lesser extent compared to the effect of anti-HLA DSA (30). Endothelium damage, microvascular inflammation, and C4d deposition on PTC endothelium are the hallmark also of ABMR mediated through anti-MICA DSA (33).

#### Non-HLA nor-MICA endothelium-restricted antigens

Along with MICA, other non-HLA systems are thought to add to the gamut of the traditional transplantation Ag (34). Indeed, ABMR may exceptionally occur following renal transplantation between non-AB0i HLA-identical siblings, who usually also share MICA, being them in *linkage disequilibrium* with the HLA genes (28).

Between 1997 and 2005, endothelium-restricted antigens (EA), expressed neither by lymphocytes nor by monocytes, were proposed as possible targets of pathogenic Ab in renal recipients who had experienced acute ABMR without any obvious DSA (35–40). Following these results, the suggestion to adopt newer cross-matching techniques that would investigate the presence of these anti-endothelial cell antibodies (AECA) in the recipient’s serum before transplantation has become stronger (41–46). Meanwhile, the first studies have come out and shown an association between circulating AECA and acute rejection, chronic rejection, poor renal graft survival, and transplant glomerulopathy (47–51).

As for the identity of EA, it is still ill defined, despite the application of proteomic, protein microarrays, and transcriptome measures (52). The most relevant information we have is that EA are expressed only by activated or damaged EC (53). This observation has lead some authors to hypothesize that the EA, or at least some of them, might actually be self-molecules rather than allo-antigens, and AECA would be auto-Ab that arise following the exposure of these cryptic self-Ag on EC primarily hit by host immune response, and they would cooperate to graft destruction with allo-immunity (54, 55). Two examples of self-Ag displayed on EC and targeted by host immune response following transplantation are angiotensin II type 1 receptor and vimentin.

##### Angiotensin II type 1 receptor

In 2005, Dragun et al. linked the presence of anti-angiotensin II type 1 receptor (AT1R) Ab to acute rejection of non-AB0i kidneys in 16 recipients without anti-HLA or anti-MICA DSA (56). Anti-AT1R Ab were studied because malignant hypertension was part of the clinical picture in all the rejecting patients, thus somehow resembling preeclampsia, a condition the researchers had already linked to the presence of such Abs (57). AT1R is present on EC and vascular smooth muscle cells and, upon ligation of angiotensin II, elicits transduction of secondary signals that contribute to the regulation of body liquids and blood pressure. Anti-AT1R Ab are agonistic non-complement-fixing immunoglobulins that promote malignant hypertension by over-activating AT1R (47); moreover, anti-AT1R Ab can induce microvascular inflammation and coagulation by stimulating NF-kB pathway and tissue factor expression by EC (58). In accordance with these data, graft biopsies from the renal recipients in Dragun’s study lacked C4d deposition, but revealed the presence of endoarteritic lesions, fibrinoid necrosis, and thrombi. The authors finally provided evidence of the pathogenic role of anti-AT1R Ab as their removal with plasmapheresis and selective blockade of AT1R with losartan significantly improved graft survival (56). The origin of such Abs is not clear yet, but, as no polymorphism of the AT1R gene has been identified, they could be auto-Ab occurring due to molecular mimicry or to anomalous presentation of over-expressed AT1R on damaged EC (58).

##### Vimentin

Vimentin is a cytoskeleton intermediate filament protein present within the cytosol of cells of mesenchymal origin, such as EC, fibroblasts, and leukocytes. Anti-vimentin auto-Abs (AVA) are described in a number of autoimmune diseases (59). In organ transplantation, exposition of vimentin isoforms on apoptotic EC, irreparably damaged by the allo-immune response, results in break of self-tolerance, emergence of active vimentin-specific CD8+ T-cells, and production of AVA (59, 60). AVA have been found in heart as well as kidney transplantation (61); albeit capable of fixing the complement and activating platelets (62), AVA alone are not pathogenic, they instead contribute with the allo-immune response to cause the vascular lesions typical of chronic rejection, and to accelerate the progression of atherosclerotic lesions (63).

### Focus on the Mechanisms of Rejection Toward Graft Endothelial Cells

**Figure 1:**
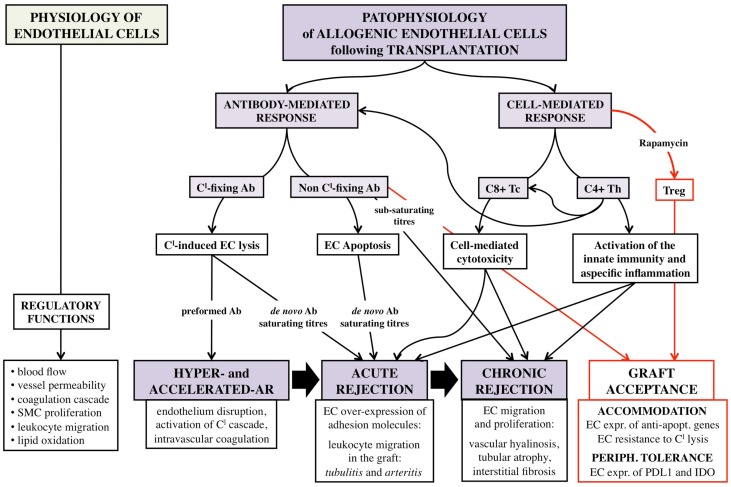
**Schematic representation of the complex interaction between allogenic endothelial cells and host immune system following vascularized solid-organ transplantation**. EC, endothelial cells; Tc, CD8+ cytotoxic T-cells; Th, CD4+ helper T-cells; Treg, CD4+ CD25+ Foxp3+ regulatory T-cells; SMC, smooth muscle cells; AR, acute rejection; Ab, antibody; C^l^, complement; expr., expression; apopt., apoptotic.

Nature and abundance of the allo-Ag expressed on EC, and type and titers of the DSA are the determinants of the intensity of host antibody-mediated immune response toward the allograft (Figure 1). This evidence has both theoretical and practice consequences. First, AB0 Ag, carbohydrates in nature, are less immunogenic than HLA molecules that are proteins, and indeed desensitization procedures for AB0i recipients are less intense and obtain better outcomes than those for anti-HLA immunized patients (16, 20). Second, A1 Ag elicit a more powerful response in kidney recipients of non-A blood group compared to A2 Ag due to minor expression of the latter on EC (13). Third, preformed DSA cause a more acute and severe rejection than *de novo* DSA, because they are more rapidly and abundantly produced by memory B-cells without any need of helper T-cells, and are more harmful for the graft (64). Fourth, complement-fixing DSA are associated with a poorer prognosis compared to non-fixing Abs (65). Finally, DSA concentration is critical for survival of both AB0i and HLAi renal transplants, with higher titers linked to more rejection episodes and shorter graft survival (20, 66).

Preformed DSA can cause hyperacute or accelerated acute rejection within minutes from the revascularization by binding to EC and fixing the complement; antibody-mediated complement activation extensively damages the endothelium integrity and initiates intravascular coagulation cascade that results in vessel thrombosis and tissue infarction (67).

Acute ABMR is a severe, albeit less catastrophic, event characterized by deposition of complement-fixing Abs on graft endothelium, mainly on the PTC endothelium, without initial activation of the coagulation (3). Upon DSA binding, activated EC increase the display on their surface membrane of MHC molecules, which further amplify the allo-immune specific response (65). Moreover, anti-HLA Ab ligation forces targeted EC to release prothrombotic mediators, like von Willebrand Factor (VWF), and to express more adhesion molecules, which foster platelets aggregation and leukocytes invasion of the graft (68). Graft invasion by T-cells is sustained by the enhanced expression on EC of vascular cell adhesion molecule-1 (VCAM-1), intercellular adhesion molecule-1 (ICAM-1), and endothelium leukocyte adhesion molecule-1 (ELAM-1) (69), adhesion molecules, which can be induced by the inflammatory mediators interleukin-1 (IL-1), tumor necrosis factor-α (TNF-α), and interferon-γ (INF-γ), produced by the lured leukocytes (70–72). The invasion by host lymphocytes and inflammatory cells of functional structures of the graft, such as the *tubuli* and the PTC, and the development of small vessel occlusion due to thrombi formation and cell accumulation determine acute deterioration of graft function (3).

Alternatively, acute episodes self-limit and relapse thereafter repeatedly, thus resulting in chronic injuries that lead to transplant atherosclerosis, tubular atrophy, and interstitial fibrosis, the hallmark of chronic rejection (24). Transplant atherosclerosis in particular is sustained by the acquisition by EC of proliferative capabilities. Upon DSA binding to HLA molecule, EC are driven to express growth factor receptors, such as the fibroblast growth factor receptor (FGF-R) (73), and to re-arrange filaments of the cytoskeleton, such as forming stress fibers (74) and recruiting integrin-ß4 (75). Cytoskeleton rearrangements confer EC the capability of reacting to appropriate stimuli by proliferating. The transduction of integrin-ß4-dependent signals activates several cytoplasmatic kinases that ultimately result in the stimulation of the mechanistic target of rapamycin (mTOR) pathway (76); mTOR activation promotes progression of cell cycle from G1 phase to S phase and ultimately induces EC division, such a proliferation is reinforced by the susceptibility to FGF of EC expressing FGF-R (73).

### Accommodation

Accommodation was originally the term used to describe the acquisition by EC of resistance to ABMR of AB0i renal grafts following reappearance of anti-A/B donor Ab (77). Nowadays, much interest is dedicated at understanding the mechanisms of accommodation and whether they could also be therapeutically harnessed to prevent transplant damage from anti-HLA DSA, that unlike anti-A/B Ab, are currently thought to invariably lead to allograft ABMR (78).

A small but interesting study investigated the behavior of EC in hyper-immune desensitized recipients who experienced reappearance of anti-HLA Abs following renal transplantation (79). None of the seven enrolled patients had hyperacute ABMR but three lost the graft due to various immunological damages, and three of the four remaining patients suffered curable acute rejection or transplant glomerulopathy. Analysis of the graft biopsies revealed increased expression of anti-apoptotic Bcl-xl gene in glomerular and peritubular capillary EC. Furthermore, *in vitro* incubation of human EC with sub-saturating concentrations of anti-HLA Ab eluted from the patients decreased ICAM-1 expression and provided resistance to complement-mediated cell lysis (79).

These preliminary results have been corroborated by further studies according to which sub-saturating anti-HLA Ab can induce EC expression of the anti-apoptotic genes Bcl-2 and Bcl-xL, whereas saturating titers induce EC apoptosis (Figure 1) (80–82).

Despite these encouraging results, a full knowledge of the accommodation process is far from been achieved and many more studies are needed in order to establish adequate protocol to desensitize hyper-immune recipients and safely perform transplantation in such population.

### Cell-Mediated Immune Response Toward Allograft Endothelial cells

As said, EC have all the properties required to drive direct activation of allogenic T-cells that is the pivotal step in all the forms of rejection non-mediated by preformed DSA (Figure 1) (8).

The rejection process has long been thought to be induced, at least initially, by donor professional APC that, upon migration into host secondary lymphoid organs, would present allo-Ag to T-cells. Nevertheless, human EC, which are not professional APC, have proven to be able to directly activate T-cells (Table 2); human EC provide “signal 1” as, unlike porcine and rodent EC, they robustly express HLA molecules, and in particular small vessel and capillary human EC constitutively express both class I and II HLA molecules (8). They also provide “signal 2” by expressing the co-stimulatory molecules required for an effective Ag presentation (8). Direct activation of T-cells by EC is of particular importance because while donor professional APC are destined to fade over time, EC, whose survival is linked to that of the allograft, can potentially ignite acute rejection at any time following transplantation (83).

**Table 2:** **Cell-mediated immune response toward allograft endothelial cells**.

T-cell types	Defining TF	Direct activation by EC	Co-stimulation (T-cell vs EC)	Outcome upon activation	Mechanisms of action	Effects on EC	Reference
CD8+ cytotoxic	–	Yes, through HLA class I	CD28 vs CD80	Graft rejection	Cytotoxicity	Enhancement of HLA expression	(8, 59, 84–86)
CD4+ helper	T-bet (Th1) GATA3 (Th2) RORγt (Th17)	Yes, through HLA class II	LFA1, LFA2 vs ICAM-1, LFA3	Graft rejection	Provision of help to B- and T-cells, guidance of innate immunity	Enhancement of HLA and adhesion molecules expression	(8, 83, 94–100)
CD4+ CD25+ Treg	Foxp3	Yes, through HLA class II	–	Tx tolerance	Disarming of APC, recruitment of new cohorts of Treg	VCAM-1 and IL-6 red.	(95–100)
						CD62E and CD62P red.	
						PDL-1 and IDO induct.	

*TF, transcription factor; EC, endothelial cells; Th, helper T-cell; Treg, regulatory T-cell; Tx, transplantation; APC, antigen-presenting cells; red., reduction; induct., induction*.

### Endothelial cells and CD8+ cytotoxic T-cells

*In vitro* mixed lymphocyte reaction (MLR) experiments using EC as stimulators of allogenic CD8+ sorted naive T-cells have confirmed that EC are able to behave as professional APC (84).

CD8+ cytotoxic T-cells (Tc), so co-cultured with EC, respond proliferating and acquiring an effector phenotype defined by higher expression of perforin and production of IL-2 and INF-γ, which in turn enhance EC expression of HLA class I and II molecules (84).

With the use of blocking monoclonal Ab (mAb), the essential signals for Tc activation have been identified in the HLA-A and B class I molecules on EC that are target of the T-cell receptor (TCR) and CD8 co-receptor, as well as the co-stimulatory molecule CD80 (B7-I) on EC and its ligand CD28 on T-cells (85). *In vivo* experiments have confirmed that CD8+ T-cell direct activation by non-hematopoietic cells, such as EC, leads to graft rejection in a murine model of class I restricted heart allo-grafts transplanted into CD4-depleted recipients devoided of secondary lymphoid organs (86). Finally, the finding of vimentin-specific autoreactive CD8+ T-cells in heart recipients have shown that transplantation cellular response, as for the humoral response, may spread from being directed to allo-antigens to autoimmunity (60).

### Endothelial cells and CD4+ helper T-cells

Accumulating evidence has convincingly clarified that human EC of microvascular origin can directly activate CD4+ helper T-cells (Th) (8, 87–89). Th are central mediators of allo-immunity as they provide help for allo-Ab production, they arm cytotoxic T-cells, and drive innate unspecific inflammatory response (8). Human EC constitutively express MHC class II molecules, HLA-DR, DP, and DQ, albeit at lesser extent compared to class I molecules. These class II molecules, which are recognized by TCR and bound with the help of CD4 co-receptor, are “signal 1” for Th direct activation. EC can also provide “signal 2” specific for Th; they indeed display ICAM-1 (CD54) and lymphocyte function-associated antigen-3 (LFA3) (CD58), which are bound by LFA1 (CD11a/18) and LFA2 (CD2) on T-cells. As shown *in vitro*, following EC provision of signals 1 and 2, Th start to proliferate and acquire an effector phenotype characterized by the induction of the co-stimulatory molecule CD40L (90) and of the adhesion molecules that favor trans-endothelium migration (91). Depending on local cytokine microenvironment, resting CD4+ T-cells differentiate into different Th subsets. Along with Th1 and Th2 subsets, whose ability to mediate rejection is well known (92), also Th17, which are implicated in a number of autoimmune diseases, can emerge guided by activated EC that provide the critical cytokine IL-6 in the presence, under inflammatory conditions, of transforming growth factor-ß (TGF-ß) (93). This is of particular interest as not only Th17 have recently proven capable of causing allograft rejection (94) but also they and CD4+ CD25+ Foxp3+ regulatory T-cells (Treg) seem to keep reciprocally at bay. Since Treg, that are crucial cells for transplantation tolerance, require TGF-ß but not IL-6 for their induction, it has been hypothesized that EC could also mediate the induction of peripheral Treg.

### Endothelial cells and regulatory T-cells

CD4+ CD25+ Treg are a well-defined subset of CD4+ T-cells identified by the expression of the master transcription factor Foxp3. They are crucial regulators of the immune response; natural Treg of thymic origin prevent autoimmune diseases, while peripherally induced Treg actively regulate transplantation tolerance (95). Treg act at a tissue level where they influence APC ability of presenting Ag to conventional T-cells (Tconv), which in turn become either anergic or regulatory cells themselves. Therefore, empowering Treg at the expense of Tconv can induce a state of local immune privilege that promotes long-term graft survival (95).

A few papers have investigated the ability of EC to interact with Treg. INF-γ-stimulated EC have proven to be capable of inducing Treg when co-cultured with allogenic CD4+ T-cells (96); but more importantly, similar results have been obtained when EC were pre-treated with the clinically available immunosuppressant rapamycin (Rapa), which exerts its functions by inhibiting the mTOR pathway (97). Expansion of Treg results from the conversion of naive CD4+ cells into Foxp3+ cells, and depends on cell–cell contact and the local microenvironment (98); not only Rapa reduces the display of VCAM-1 in EC (99) but also forces the expression of the inhibitory molecules programed death ligand-1 (PDL-1) and indoleamine 2,3-dioxygenase (IDO), which are crucial for Treg induction (96–98). Finally, EC pre-treated with Rapa produce less IL-6 (97), which is instead required for the expansion of Th17 but not that of Treg (93). EC-induced Treg are functionally active as they can effectively suppress the proliferative response of Ag-stimulated CD8+ T-cells. On the other side, Treg can influence EC behavior; Treg release TGF-ß that downregulate the expression on EC of the adhesion molecules CD62E and CD62P (respectively, E- and P-selectin), thus limiting transmigration of Tc and reducing local inflammation (100).

## Conclusion

Graft endothelium is the first barrier between self and non-self in transplantation of vascularized solid organs, such as kidney transplants. Indeed, upon organ reperfusion, host lymphocytes initially encounter graft EC, which express all the most relevant antigens in transplantation immunobiology. Such antigens are invariably recognized by the host immune system, and toward them antibody-mediated and cell-mediated immune responses mount. Moreover, not only are EC a passive target of the host immune system but they also are an active player in the recruitment and activation of allogenic lymphocytes, and in the invasion of graft tissues by them. The ability of EC to directly activate allogenic naive T-cells deserves to be highlighted as EC represent a long-term source of allo-antigens and can potentially induce graft rejection at any time post-transplantation. Therefore, the fate of renal transplants largely depends on how such interplay between graft endothelium and host immune system unfolds: on one side, the activation of the immune response may lead to all the forms of graft rejection, from hyperacute to chronic, and to the deterioration of graft function primarily because of damages to the endothelium integrity. On the other side, even in the presence of circulating donor-specific antibodies directed to antigens expressed on donor EC, graft endothelium may thrive and fulfill its functions of regulating blood flow and vessel permeability, which are crucial not only for graft survival but also for the depurative activities of the kidney.

Current immunosuppressive drugs have been developed with the aim of targeting host immune response, be it depleting or blocking T-cells and B-cells or halting the complement cascade; however, given the central role of EC in modulating the allo-immune response, graft endothelium may represent a preferential target of newer immunosuppressive protocols aimed at promoting long-term graft acceptance by reducing EC immunogenicity and antigen presentation, while favoring their survival. The class of immunosuppressants mTOR inhibitors has been more and more utilized in the clinic for its pro-tolerogenic and anti-neoplastic activities; the pro-tolerogenic effects of the mTOR inhibitor rapamycin have proven to rely at least in part on its ability to condition antigen presentation by EC so that regulatory T-cells emerge at the expense of rejecting conventional T-cells. Although scientific data lack, it is conceivable that also the latest immunosuppressant introduced in the clinic, belatacept, may prevent graft rejection by limiting EC ability to activate T-cells (101); belatacept is a CTLA-4 fusion protein with the human IgG Fc, which blocks “signal 2,” by binding to the co-stimulatory molecules CD80 and CD86 that are expressed not only on the APC but also on the surface of EC.

As therapeutically blocking EC from presenting antigens prevents direct activation of T-cells and, in turn, induction of *de novo* donor-specific antibodies, developing such immunosuppressive strategy could become the answer to the issue of late ABMR, toward which current immunosuppressants are blunt, and could contribute to the improvement of long-term transplantation outcomes.

## Conflict of Interest Statement

The authors declare that the research was conducted in the absence of any commercial or financial relationships that could be construed as a potential conflict of interest.
